# Erratum

**Published:** 2005-06

In Figures 1, 2, and 3 of “Altered Profiles of Spontaneous Novelty Seeking, Impulsive Behavior, and Response to d-Amphetamine in Rats Perinatally Exposed to Bisphenol A” by Adriani et al. [ Environ Health Perspect 111:395–401 (2003)], results for oil controls and bisphenol A (BPA)-treated rats were labeled incorrectly. The corrected figures are shown below. *EHP* apologizes for the errors.

## Figures and Tables

**Figure 1:**
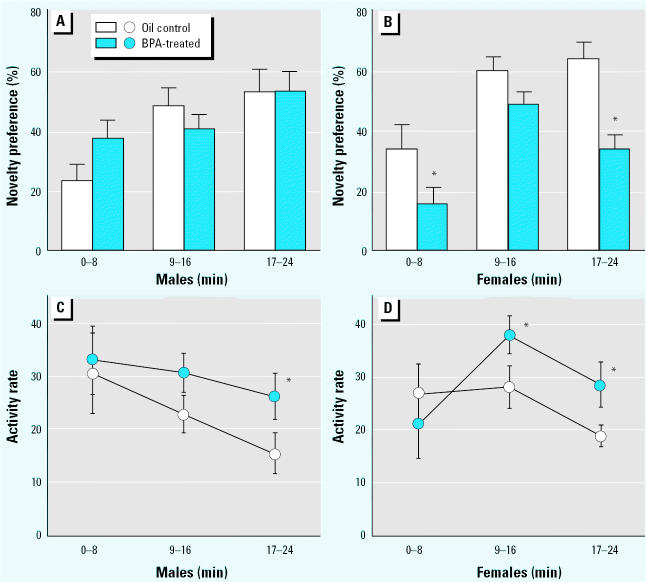
(*A,B*) Mean (± SE) percentage of time spent in the novel compartment by subjects of both sexes on testing day (experiment 1). (*C,D*) Mean (± SE) activity rate, measured as number of line crossings per minute, shown by subjects of both sexes in the novel compartment on testing day. During the pretreatment period (days 1–3), subjects were familiarized to one compartment. On testing day, animals were placed in the familiar compartment. After 5 min, a partition was removed and subjects were allowed free access to a novel compartment of the apparatus for a 24-min session.
*p < 0.05 in comparisons between BPA and control perinatal treatments (*n* = 9).

**Figure 2:**
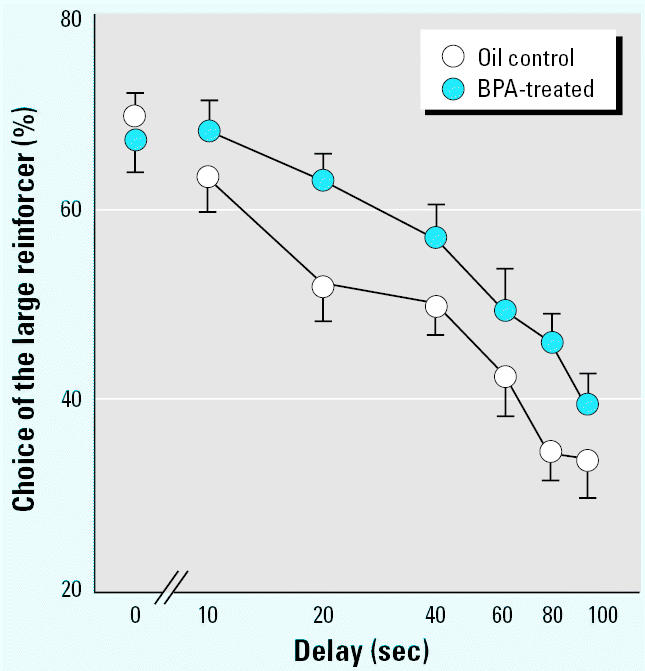
Mean (± SE) choice (%) of the large reinforcer, demanded by nose poking at the LAD hole, shown by rats during the test for impulsivity (experiment 2). These data reveal that, as the length of the delay increased, animals increased demanding the small but immediate reinforcement and decreased demanding the larger but delayed one. A shift to the right of the whole curve (i.e., a profile of reduced impulsivity) was evident in BPA-exposed rats compared with controls. In the absence of significant differences, data from the two sexes were collapsed (*n* = 18).

**Figure 3:**
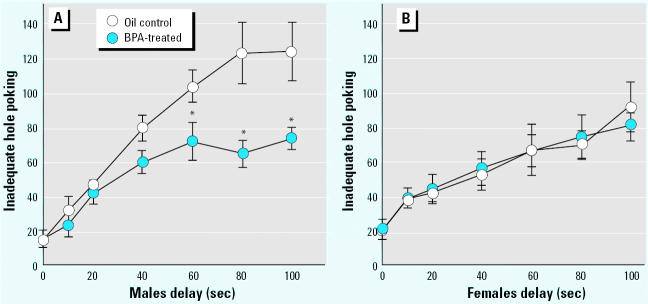
Mean (± SE) frequency of inadequate responding at the IAS hole (i.e., nose poking during the length of the delay, when it was without any consequence) shown by rats during the test for impulsivity (experiment 2). These data reveal that, when animals were waiting for the delivery of the large reinforcer, they failed to rest and were demanding the immediate one. A clear-cut demasculinization in the restlessness profile was evident.
*p < 0.05 in multiple comparisons between BPA and control perinatal treatments (*n* = 9).

